# Cumulative incidence and predictors of acquired aortic stenosis in a large population of men followed for up to 43 years

**DOI:** 10.1186/s12872-022-02487-y

**Published:** 2022-02-13

**Authors:** Silvana Kontogeorgos, Erik Thunström, Georgios Lappas, Annika Rosengren, Michael Fu

**Affiliations:** 1 Department of Molecular and Clinical Medicine, Institute of Medicine, Sahlgrenska Academy, University of Gothenburg, Gothenburg, Sweden; 2 Region Västra Götaland, Sahlgrenska University Hospital/Östra Hospital, Diagnosvägen 11, 416 50 Gothenburg, Sweden

**Keywords:** Aortic stenosis, Obesity, Cumulative incidence, Predictive factors

## Abstract

**Background:**

Acquired aortic stenosis (AS) increases with age and has high mortality without intervention. Factors predicting its development are unclear, although atherosclerotic factors are assumed to be involved. Our aim in this study is to estimate the lifetime cumulative incidence and predictors of AS in middle-aged men.

**Methods:**

We included a random sample of men (n = 9998) born 1915–1925 in Gothenburg, Sweden. From them, 7,494 were examined and followed until a diagnosis of AS or death (maximum follow-up time 42.8 years). We identified AS diagnosis from the Swedish National Patient Registry and deaths from the Swedish Cause of Death Registry by using International Classification of Disease (ICD) diagnostic criteria. To study time-dependent relationships between AS and risk factors with death as the competing risk, we divided the cohort into three overlapping follow-up groups: 25–43, 30–43 and 35–43 years. We used age-adjusted Cox proportional hazards model to identify predictors of AS.

**Results:**

The lifelong cumulative incidence of AS was 3.2%. At baseline, participants in the third group had a healthier lifestyle, lower body mass index (BMI), blood pressure, and serum cholesterol levels. Higher BMI, obesity, cholesterol, hypertension, atrial fibrillation, smoking and heredity for stroke were associated with AS. With BMI of 20–22.5 as a reference, hazard ratios of being diagnosed with AS for men with a baseline BMI of 25–27.5 kg/m^2^, 27.5–30 kg/m^2^ and > 30 kg/m^2^ were 1.99 (95% CI 1.12–3.55), 2.98 (95% CI 1.65–5.40) and 3.55 (95% CI 1.84–6.87), respectively.

**Conclusions:**

The lifetime cumulative incidence of AS in middle-aged male population was 3.2%. Multiple atherosclerotic risk factors, particularly high BMI might be associated with a higher risk of developing AS.

## Background

The incidence of acquired nonrheumatic aortic stenosis (AS) increases dramatically with age [1] and, if left untreated, is associated with high mortality in symptomatic patients [2]. With rising life expectancy, AS is becoming more common and one of the most frequently occurring valvular heart disease. The pathophysiological changes leading to aortic valve sclerosis and AS result from the synergy of the mechanical forces and the changes in the tissular environment sharing similarities with changes encountered in ischemic heart disease [3]. Studies have shown an association between AS and obesity [4, 5], hypercholesterolemia [6–9] or hypertension [6, 7, 10, 11], with increasing evidence that atherosclerotic risk factors might also affect the development of aortic valve sclerosis and its progression to AS. However, another question arises as to whether the relationships mentioned above between AS and risk factors persist over the life course, considering that several of these factors are prone to change over time. This issue is of particular interest given that AS is a slowly progressive disease, generally taking decades to develop. Accordingly, we have recently shown that high body mass index (BMI) in middle aged men may predict AS after 21 years [12]. However, the sample size in that study was small. Here, we aim to determine the cumulative incidence of AS and its associated factors over a lifetime and examine the relationship between BMI and AS in a larger sample size, incorporating a more extended follow-up period.

In the past decades the prevalence of overweight and obesity has been steadily increasing, now emerging as a major health problem [13], obesity being a risk factor for a variety of diseases (e.g., diabetes, cancer, sleep apnoea) and for cardiovascular diseases in general [14]. Moreover, obesity associates with hypertension, left ventricular hypertrophy, abnormal left ventricular geometry [15], and ischemic heart disease [16, 17].

The aims of this study are to investigate the cumulative incidence of AS in a large population and to identify the factors at baseline that could be associated with the AS diagnosis.

## Study population and data collection

This study included participant men in the intervention group of the multifactor Primary Prevention Study (PPS), which began in 1970 in Gothenburg, Sweden [18]. All men (n = 30,000) born between 1915 and 1925 (except those born 1923 who were included in another study) and residing in Gothenburg were randomized into three groups: an intervention group of 9998 men and two control groups of similar size. The study had three examination periods: 1970–1973 (baseline), 1974–1977 and 1980–1983. At baseline (1970–1973), the men in the intervention group, then aged 47–55 years, were screened for cardiovascular risk factors (family history of myocardial infarction or stroke, psychological stress, smoking, total cholesterol levels, blood pressure, physical activity, diabetes, overweight) and those with high risk were treated (high blood pressure, severe hypercholesterolemia, heavy smoking). No significant differences in risk factors or outcome (cardiovascular diseases, cancer, and all-cause mortality) were detected in the first 12 years of follow-up, after which the intervention ended [19]. Accordingly, we considered that the final group of individuals, although being a part of an intervention study, represented Gothenburg's general male population.

Information about risk factors (family history, smoking, physical activity during leisure time, diabetes, self-reported weight at age 20) was obtained by questionnaire. Weight was measured in kilograms to the nearest 0.10 kg and height was measured in meters to the nearest 0.01 m. BMI was calculated as weight in kilograms divided by the square of height in meters. Obesity was defined as BMI ≥ 30 kg/m^2^. Blood pressure (BP) was measured to the nearest 2 mm Hg in the sitting position after 5 min of rest. Hypertension was defined as the use of antihypertensive medicines or systolic BP (SBP) > 160 mm Hg, or diastolic BP (DBP) > 95 mm Hg. Pulse pressure (PP) was defined as SBP minus DBP. Total serum cholesterol levels were measured after fasting for ≥ 2 h and were determined according to standard laboratory procedures. Smoking habits were divided into three categories: current smoker, non-smoker and former smoker (> 1 month). Physical activity during leisure time was categorized into a sedentary lifestyle and an active lifestyle (at least 4 h per week of physical activity). Diagnoses at screening and follow-up (hypertension, diabetes, ischemic heart disease, stroke, atrial fibrillation, heart failure, renal failure) were identified through the Swedish National Patient Registry (NPR). The diagnoses were time-updated for the period 1970–2012.

### Follow-up

All the participants were followed from their baseline examination until October 2012, a registered diagnosis of AS, or death, whichever came first. The maximum follow-up time was 42.8 years (mean 26.8 years).

### Data on aortic stenosis

We used the NPR to identify men with a registered diagnosis of AS and the Swedish Cause of Death Registry to identify those who died and from what causes, based on the personal identification number assigned to each Swedish resident. Loss due to emigration was negligible. AS was defined as a diagnosis of aortic valve disease according to the International Classification of Disease (ICD) diagnostic criteria: code 424.10 for ICD-8 (used until 1986), code 424B for ICD-9 (in use 1987–1996) or code I35.0, I35.2 for ICD-10. Individual patient records for men diagnosed before 1997 were revised manually (the same code was used to classify aortic stenosis and insufficiency in ICD-8 and ICD-9). Patients diagnosed with isolated aortic insufficiency were identified and not included in the AS group.

### Statistical analysis

Continuous variables are expressed as mean ± standard deviation or median and IQR, as appropriate. Categorical variables are presented as frequencies and percentages. Cumulative incidences of AS were estimated and presented in diagrams for some baseline factors accounting for death as competing risk.

To study the time-dependent relationship between risk of AS and baseline factors and control in some degree the effect of the baseline factors on the competing risk of death, the follow-up period was divided into three overlapping time intervals: follow-up between 25 and 43 years, 30 and 43 years and the third group, including the individuals who survived longest, with follow-up between 35 and 43 years.

The associations between the risk of AS and each one of the actual baseline factors and time-updated comorbidities were estimated with the help of a hazard ratio calculated with Cox proportional hazards regression model, where age and the relevant factor were covariates. Although the continuous baseline factors when entered in the Cox model as cubic splines result in a better, albeit marginally, fit (due to possible nonlinear relationship between the factors and the risk of AS), we choose to present the mean (linear) effect of the actual factor over the range of its values and for the actual follow-up period as a summary measurement of the effect on AS risk. While we did not adjust for risk factors that are caused by overweight/obesity such as diabetes, hypertension and dyslipidaemia, we added a second model where we adjusted for sedentary behaviour, baseline smoking and family history.

The statistical analyses were performed using *SAS software version 9.2 (SAS Institute Inc, Cary, North Carolina)* and *R 3.6.2 *[20]*.*

## Results

### Cumulative incidence of AS

**Fig. 1:**
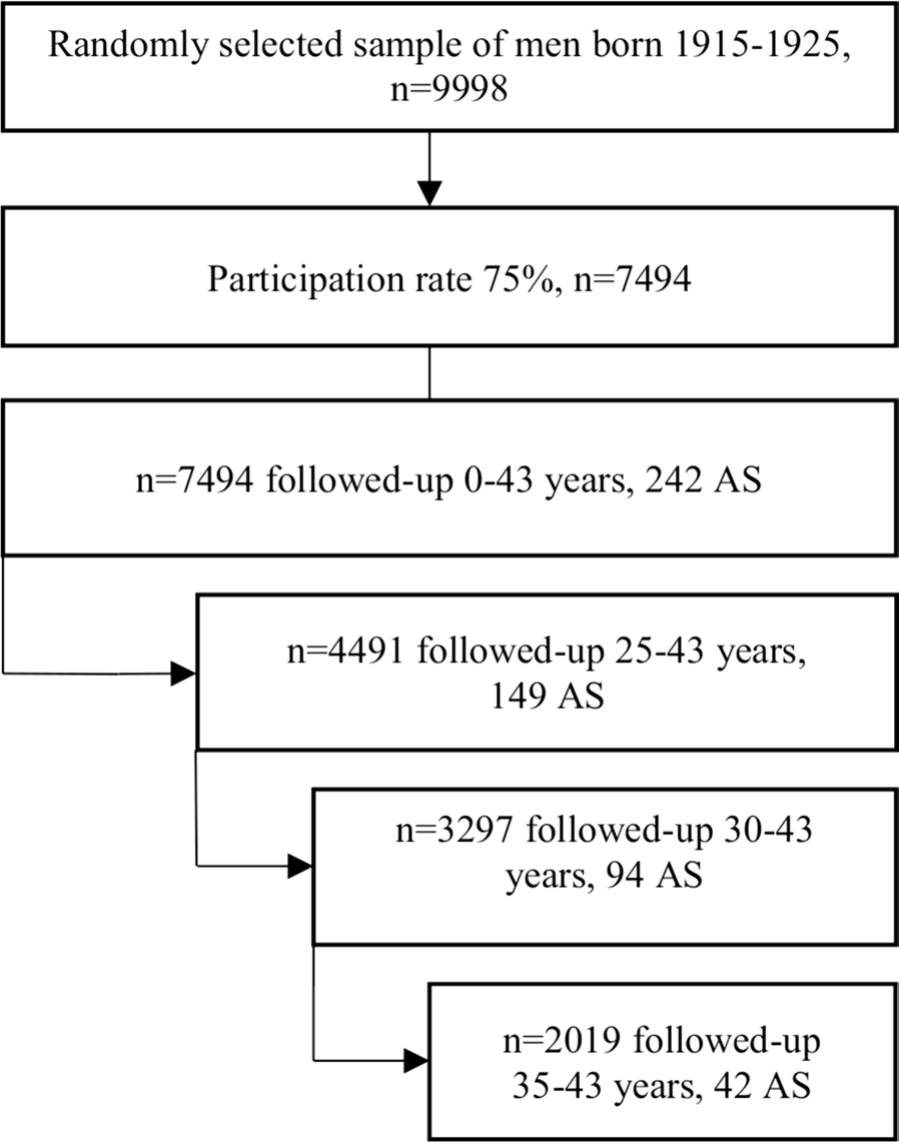
Flow chart of the study population

As shown in Fig. 1, the participation rate was 75% (7494 individuals). They were followed from the inclusion date until death or October 2012. The mean follow-up time was 27 years and the maximum follow-up was 43 years. The mean age of the participants at baseline was 51 years. Overall cumulative incidence of AS was 3.2% (242/7494 individuals) over the follow-up period. The corresponding rates in the three subgroups were 3.3% (149/4491), 2.9% (94/3297) and 2.1% (42/2019) for follow-ups between 25 and 43 years, 30 and 43 years and 35 and 43 years, respectively.

### Baseline characteristics

Baseline and follow-up characteristics of the participants are described in Table 1. Individuals in the group surviving ≥ 35 years were characterized by the comparatively lower weight and BMI at screening and lower weight gain from age 20. They were also healthier at baseline, with lower BP, PP, and total cholesterol values. They also had a healthier lifestyle with a lower current smokers’ rate and fewer living a sedentary lifestyle. In addition, these participants had a more favourable heredity profile, with a lower percentage of stroke or myocardial infarction in parents and siblings. Table 1 presents comorbidities both at baseline (in the group followed up from 0 to 43 years) and time-updated. The comorbidities with the highest prevalence were found in the individuals with the longest follow-up (35–43 years).

**Table 1:** Baseline levels and prevalence of risk factors of the study population at four overlapping periods shrinking towards the end of follow-up

Follow-up interval	0–43 years	25–43 years	30–43 years	35–43 years
Number of patients, n	7494	4491	3297	2019
Aorta events n (%)	242 (3.2%)^1^	149 (3.3%)^1^	94 (2.9%)^1^	42(2.1%)^1^
*Follow up (years)*				
Mean (SD)	26.86 (10.50)^1^	9.09 (5.08)^1^	6.46 (3.63)^1^	3.97 (2.00)^1^
*Baseline levels and factors at screening 1970–1973*				
Age (years)				
Mean (SD)	51.07 (2.28)^1^	50.80 (2.30)^1^	50.56 (2.30)^1^	50.25 (2.28)^1^
Weight (kg)				
Mean (SD)	78.85 (11.37)^3^	78.66 (10.62)^2^	78.60 (10.44)^1^	78.50 (10.26)^1^
Weight gain, 30 (kg)				
Mean (SD)	9.84 (9.47) ^3^	9.52 (8.79) ^2^	9.30 (8.53)^1^	9.05 (8.19)^1^
Missing	467	245	162	87
BMI (kg/m^2^)				
Mean (SD)	25.54 (3.27)^3^	25.42 (3.01)^2^	25.37(2.95)^1^	25.28 (2.85)^1^
Obesity (BMI ≥ 30 kg/m^2^), n (%)	617 (8.2%)^3^	296 (6.6%)^2^	203 (6.2%)^1^	113 (5.6%)^1^
Systolic blood pressure, mmHg				
Mean (SD)	148.77 (21.94)	146.37 (21.08)	145.18 (20.50)	143.75 (20.08)
Missing	12	4	2	1
Diastolic blood pressure, mmHg				
Mean (SD)	94.64 (12.94)	93.40 (12.43)	92.76 (12.30)	92.08 (12.01)
Missing	13	5	2	1
Heart rate, beats/min				
Mean (SD)	73.21 (13.53)	71.99 (12.86)	71.41 (12.59)	70.96 (12.86)
Missing	40	19	8	4
Pulse pressure, mmHg				
Mean (SD)	54.14 (14.82)	52.96 (14.30)	52.41 (13.96)	51.67 (13.82)
Missing	14	5	2	1
Cholesterol, mmol/L				
Median (IQR)	6.32 (1.46)	6.32 (1.33)	6.32 (1.46)	6.21 (1.46)
Missing	78	29	15	9
Smoking, n (%)				
Never	2201 (29.4%)^1^	1546 (34.4%)^1^	1222 (37.1%)^1^	837 (41.5%)^1^
Current	3768 (50.3%)^1^	1923 (42.8%)^1^	1302 (39.5%)^1^	674 (33.4%)^1^
Former	1524 (20.3%)^1^	1021 (22.7%)^1^	772 (23.4%)^1^	507 (25.1%)^1^
Sedentary life, n (%)	1919 (25.6)^1^	1002 (22.3)^1^	701 (21.3)^1^	399 (19.8)^1^
Family history of myocardial infarction, parents^1^ n (%)	1505 (20.1%)^1^	859 (19.1%)^1^	618 (18.7%)^1^	378 (18.7%)^1^
Family history of stroke, parents^1^ n (%)	440 (5.9%)^1^	216 (4.8%)^1^	141 (4.3%)^1^	71 (3.5%)^1^
Family history of myocardial infarction, siblings^1^ n (%)	1989(26.5)	1152(25.7)	815(24.7)	473(23.4)
Family history of stroke, siblings^1^ n (%)	196(2.6)	96(2.1)	67(2.0)	38(1.9)
*Time-updated diagnoses 1970–2012*				
Hypertension n (%)	3690 (49.2%)^1^	2098 (46.7%)^1^	1576 (47.8%)^1^	1030 (51.0%)^1^
Diabetes n (%)	149 (2.0%)^1^	285 (6.3%)^1^	270 (8.2%)^1^	200 (9.9%)^1^
Ischemic heart disease n (%)	1131 (15.1%)^1^	1232 (27.4%)^**1**^	1014 (30.8%)^**1**^	681 (33.7%)^**1**^
Stroke n (%)	43 (0.6%)^**1**^	318 (7.1%)^**1**^	362 (11.0%)^**1**^	262 (13.0%)^**1**^
Atrial fibrillation n (%)	4 (0.1%)^**1**^	376 (8.4%)^1^	481 (14.6%)^1^	391 (19.4%)^1^
Renal failure n (%)	1 (0.0%)^1^	427 (9.5%)^1^	524 (15.9%)^1^	419 (20.8%)^1^
Heart failure n (%)	0 (0.0%)^**1**^	302 (6.7%)^1^	375 (11.4%)^1^	291 (14.4%)^1^

*SD* standard deviation, *IQR* interquartile range, *BMI* body mass index

Missing data: ^1^n = 1, ^2^n = 2, ^3^ n = 3

### Association of AS with baseline factors

In age-adjusted analyses (Table 2) baseline body weight (per kg) at screening was significantly associated with the development of AS in the whole cohort (HR = 1.02; 95% CI 1.01–1.03 per kg) and in the group followed from 25 to 43 years (HR = 1.02; 95% CI 1.01–1.04 per kg). BMI as a continuous variable was associated with AS in all groups, except the group followed from 30 to 43 years, with the highest HR of 1.12 (95% CI 1.01–1.24) per BMI unit in the group with the longest survival. Obesity was also associated with AS: the baseline HR = 1.64 (95% CI 1.08–2.51), increasing to 1.83 (95% CI 1.07–3.12) in the group followed up 25–43 years and to 3.13 (95% CI 1.31–7.45) in men surviving 35 years. There was also an association between serum cholesterol and AS in the whole cohort, HR = 1.26 (95% CI 1.15–1.39) per mmol/L. For participants followed 25–43 years, the HR was 1.29 (95% CI 1.15–1.46) and 1.22 (95% CI 1.04–1.43) for those followed 30–43 years. There was no association between serum cholesterol and AS in those surviving the longest.

**Table 2:** Age-adjusted Cox regression hazard model

Follow-up interval	0–43 years	25–43 years	30–43 years	35–43 years
*Screening age-adjusted HR (95% CI)*				
BMI (per BMI unit)	1.08 (1.04–1.12)	1.09 (1.03–1.15)	1.07 (1.00–1.15)	1.12 (1.01–1.24)
Weight at screening (per kg)	1.02 (1.01–1.03)	1.02 (1.01–1.04)	1.02 (1.00–1.04)	1.03 (1.00–1.06)
Weight gain, 30 years (per kg)	1.02 (1.01–1.03)	1.02 (1.00–1.04)	1.01 (0.99–1.04)	1.03 (0.99–1.07)
Obesity (yes/no)	1.64 (1.08–2.51)	1.83 (1.07–3.12)	1.62 (0.78–3.35)	3.13 (1.31–7.45)
*Smoking*				
Former vs never, Current vs never	1.09 (0.76–1.54) 1.32 (0.99–1.77)	1.35 (0.88–2.08) 1.54 (1.05–2.25)	1.22 (0.69–2.14) 1.89 (1.18–3.03)	0.70 (0.27–1.82) 2.08 (1.07–4.07)
Sedentary lifestyle (yes/no)	0.84 (0.61–1.16)	0.77 (0.50–1.18)	0.81 (0.47–1.38)	1.10 (0.53–2.30)
Systolic blood pressure (per mm Hg)	1.01 (1.00–1.01)	1.01 (1.00–1.02)	1.01 (1.00–1.02)	1.00 (0.99–1.02)
Diastolic blood pressure (per mm Hg)	1.01 (1.00–1.02)	1.01 (1.00–1.03)	1.01 (0.99–1.02)	1.01 (0.98–1.03)
Pulse pressure (per mm Hg)	1.01 (1.01–1.02)	1.01 (1.00–1.02)	1.02 (1.00–1.03)	1.00 (0.98–1.02)
Heart rate (beats/min)	1.01 (1.00–1.02)	1.01 (1.00–1.02)	1.01 (0.99–1.02)	1.00 (0.97–1.02)
Family history of myocardial infarction, parents (yes/no)	1.33 (0.99–1.79)	1.00 (0.66–1.50)	0.89 (0.52–1.52)	1.03 (0.48–2.23)
Family history of stroke, parents (yes/no)	1.14 (0.86–1.51)	1.23 (0.86–1.76)	1.38 (0.89–2.15)	1.88 (1.00–3.53)
Family history of myocardial infarction, siblings (yes/no)	2.06 (1.33–3.20)	2.59 (1.52–4.42)	1.43 (0.58–3.52)	0.00 (0.00-Inf)
Family history of stroke, siblings (yes/no)	2.02 (1.07–3.80)	2.86 (1.40–5.84)	3.61 (1.58–8.27)	4.35 (1.34–14.09)
Cholesterol (per mmol/L)	1.26 (1.15–1.39)	1.29 (1.15–1.46)	1.22 (1.04–1.43)	1.07 (0.82–1.40)
Atrial fibrillation (yes/no)	-	2.00 (1.19–3.37)	2.25 (1.37–3.69)	2.30 (1.19–4.44)
Diabetes (yes/no)	1.57 (0.50–4.90)	1.19 (0.56–2.54)	1.06 (0.46–2.44)	1.11 (0.40–3.12)
Hypertension (yes/no)	1.40 (1.09–1.80)	1.57 (1.14–2.17)	1.86 (1.23–2.82)	1.77 (0.95–3.31)
Ischemic heart disease (yes/no)	1.54 (1.09–2.18)	1.47 (1.03–2.08)	1.61 (1.06–2.45)	1.49 (0.80–2.75)
Stroke (yes/no)	1.49 (0.21–10.63)	0.60 (0.22–1.62)	0.80 (0.35–1.83)	1.66 (0.74–3.75)
Heart failure (yes/no)	–	1.20 (0.53–2.74)	1.81 (0.96–3.42)	2.69 (1.32–5.48)
Renal failure (yes/no)	–	8.83 (2.79–27.92)	1.33 (0.18–9.55)	0.00 (0.00-Inf)

*BMI* body mass index, *CI* confidence interval, *HR* hazard ratio

**Fig. 2:**
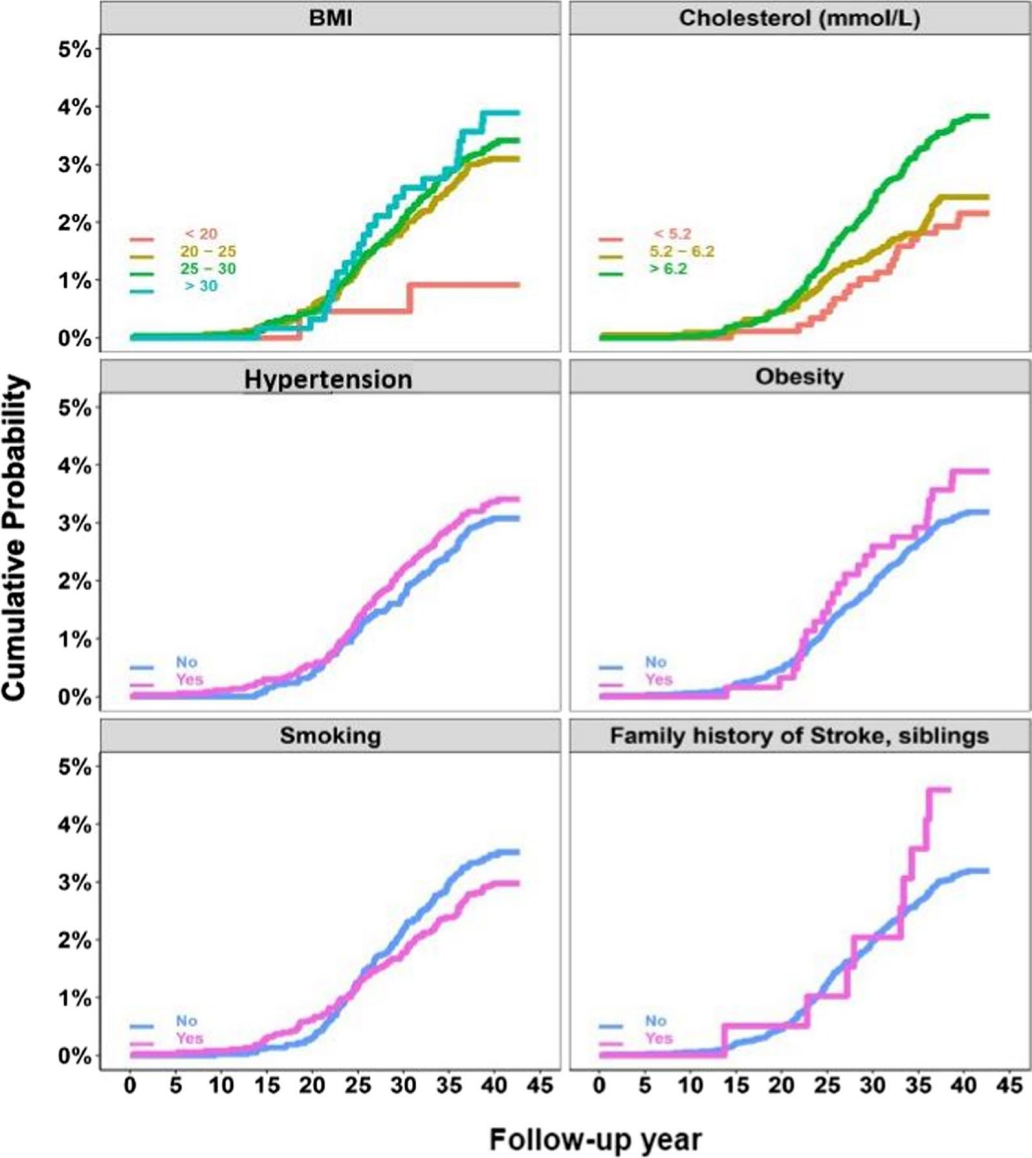
Cumulative incidence of AS according to categorical baseline factors

Having a sibling with stroke was also associated with the risk of developing AS during follow-up, retaining its predictive power over all groups, but with relatively wide confidence intervals. The highest HR was observed in the third group (i.e. with the longest survival time), HR = 4.35 (95% CI 1.34–14.09). An association was found in comparing current smokers with never smokers in all but the group followed from 0 to 43 years, with the highest HR of 2.08 (95% CI 1.07–4.07) in the group with the longest survival time. Hypertension or ischemic heart disease during follow-up were associated with AS in the whole cohort, as well as in the first and middle groups, but not in the group with the longest survival time. Participants with atrial fibrillation had a higher risk of being diagnosed with AS during the follow-up time. The risk increased with more prolonged survival, with the highest HRs in those surviving ≥ 35 years (HR = 2.30; 95% CI 1.19–4.44). Renal failure was also associated with AS, but only in the individuals followed-up 25–43 years. However, the wide confidence intervals reflects a low statistical precision in this case. The cumulative incidence of AS according to the factors significantly associated with the AS diagnosis is depicted in Fig. 2.

**Fig. 3:**
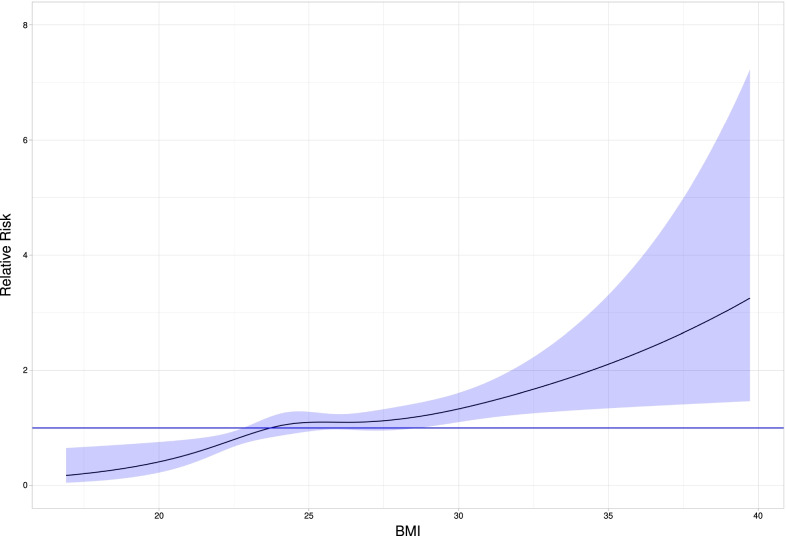
Restricted cubic spline estimate of the relationship between BMI and relative risk for AS

Figure 3 shows the age-adjusted association between baseline BMI and AS, with a continuous increase from low levels up to about 30 kg/m^2^; at higher BMI levels, confidence intervals were wide. Using a BMI between 20 and 22.5 kg/m^2^ as the reference category, overweight men with a BMI from 25 to 27.5 kg/m^2^ from the whole cohort had a 1.99 higher risk of developing AS (95% CI 1.12–3.55, p = 0.019). The risk was 2.98 (95% CI 1.65–5.40 p < 0.001) in the category 27.5–30 kg/m^2^ and 3.55 in obese men (BMI ≥ 30 kg/m^2^) (95% CI 1.84–6.87, p < 0.001) (Table 3), after adjusting for age. Results were essentially unchanged after further adjustment for sedentary behaviour, baseline smoking and family history.

**Table 3:** Hazard ratios risk for AS for the whole cohort relative to the BMI with reference category of 20.0–22.5 adjusted for (1) age (model 1), (2) age, baseline smoking, sedentary activity and family history of stroke or myocardial infarction

	Model 1		Model 2	
	Hazard ratio (95% CI)	P value	Hazard ratio (95% CI)	P value
BMI < 20.0 kg/m^2^	0.93 (0.21–4.10)	0.92	0.88 (0.20–3.88)	0.87
BMI 20–22.5 kg/m^2^	1 (reference)	-	-	-
BMI 22.5–25.0 kg/m^2^	2.48 (1.41–4.37)	< 0.002	2.53 (1.44–4.45)	< 0.001
BMI 25.0–27.5 kg/m^2^	1.99 (1.12–3.55)	0.019	2.07 (1.16–3.69)	0.010
BMI 27.5–30.0 kg/m^2^	2.98 (1.65–5.40)	< 0.001	3.17 (1.75–5.74)	< 0.001
BMI > 30 kg/m^2^	3.55 (1.84–6.87)	< 0.001	3.83 (1.98–7.43)	< 0.001

*BMI* body mass index

## Discussion

In this large population of middle-aged men followed up to 43 years, 242/7494 men (or 3.2%) were diagnosed with AS. Multiple factors that heighten the risk of atherosclerosis were also linked to an increased risk of developing AS.

Few studies have assessed the cumulative incidence of AS from middle age, where prevalence is low, up to advanced age. Many studies have reported prevalence rates based on echocardiographic examinations by age group. For instance, rising rates from 1.3% between 60 and 69 and 3.9% between 70 and 79 years have been reported in the Tromsø study [1]. Another study based on three cohorts of randomly selected US adults from the general population reported an increase from 0.7% in 18–44-year-olds to 13.3% in the age group ≥ 75 [21]. In addition, the estimated prevalence may vary greatly depending on the criteria used [7, 22]. In this study we investigated a population diagnosed with AS in the hospital using registered diagnostic codes. In contrast, most of the prevalence studies used echocardiographic-verified AS in the general population. Our results are therefore not directly comparable. Some studies have shown that male sex could be a risk factor for AS [7, 21, 23]. Thus, the cumulative incidence of our cohort containing only men may be overestimated.

The second goal of this study was to identify factors at baseline or during follow-up that could predict individuals with an elevated risk of developing AS during their lifetime. The extended follow-up of our study participants (42.8 years) poses problems in studying a slowly progressive disease such as AS, with death as a competing event. To address this issue we studied the cohort in three overlapping time intervals. In the first interval, the individuals still alive after 25 years and free of AS were followed until the end. Similar follow-up was conducted for the intervals 30 and 35 years after the baseline as start point. In this manner, we wanted to determine whether the baseline factors or the time-updated comorbidities associated with the incidence of AS in the whole follow-up interval were still important with increasing longevity, retaining the predictive power or even increasing in effect (e.g. the variables “obesity” and “smoking”), due to association of these factors with the competing risk of death.

We found that obese individuals at baseline had a 64% higher risk of receiving a diagnosis of AS in the whole cohort. This risk increased to 83% in the first group and was three times higher in the longest surviving participants, but not in the intermediate group, although estimates were numerically similar. The spline function suggested a curvilinear association between BMI and AS; however, because few men were obese at baseline, the effect of severe obesity could not be investigated in our sample. Yet, our results provide some data and help clarify this issue, which has been controversial. Some studies showed a positive association between AS and obesity, including a Swedish study using patient registries [24], a large case–control study with older patients with severe calcified AS [4], and a recent Danish study [5]. Three more studies showed a positive correlation between BMI and aortic valve calcification [8, 25, 26], whereas three others did not show such a correlation. A substudy of SEAS (Simvastatin Ezetimibe in AS) failed to find an association between AS and obesity in multivariable analysis. Still, in this study, all the patients who had CAD, diabetes mellitus, peripheral arterial disease, cerebrovascular disease or other indication for treatment with statins were excluded [15]. No link was found either between weight and aorta valve calcification in a small cross-sectional study [27]. A larger, cross-sectional study of 5,201 patients ≥ 65 years enrolled in the Cardiovascular Health Study, where aortic sclerosis and AS were considered together also failed to observe an association between weight and aorta valve calcification [7]. However, in this study, < 10% of those with aortic disease were defined as AS and, in addition, the cross-sectional design does not permit to investigate the effect in time of obesity on AS. Finally, another study, in an elderly, unselected population, lower BMI (leanness) was associated with a higher risk for aortic valve calcification [10]. Accordingly, although aortic non-stenotic disease may progress to stenosis, risk factors for aortic sclerosis or calcification may differ from those associated with AS progression. In clinical studies AS patients with obesity [28] or metabolic syndrome [29, 30] had a more accelerated AS progression, suggesting that BMI over the normal limit might play an important role in AS physiopathology.

A possible explanation for the effect of high BMI on the development or progression of AS might be that overweight/obesity is associated with other risk factors of atherosclerosis (such as diabetes, hypercholesterolemia, hypertension) that might lead to the incipient phase of the process of valve degeneration [3]. In addition, the inflammatory state accompanying obesity [31] may play a role in the development of early lesions on the aortic valves, lesions characterized by an active inflammatory process [3].

Hypercholesterolemia is a key factor in the development of atherosclerotic plaque and coronary artery disease. Similarly, lipid accumulation has been shown in early lesions appearing on degenerative aortic valves [3]. Therefore, it is expected that high cholesterol levels would increase the risk of AS development. However, findings on the relationship between hypercholesterolemia and AS development or progression are inconsistent and questionable. In our study, cholesterol level was associated with the risk of being diagnosed with AS in the whole cohort, but not when those surviving ≥ 35 years were analyzed separately. The higher mortality risk may explain that the association is not significant in this last, most “healthy” group. These individuals (now in their 80 s) with higher cholesterol levels have less chance of surviving and be diagnosed with AS after 35 years of follow-up.

Hypertension is often associated with degenerative AS and portends worse prognosis and faster progression [32]. Our study found that hypertension was associated with being diagnosed with AS, similar to previous associations between hypertension and AS [7, 10, 11] or aortic sclerosis [6, 33]. Potential explanations for the association of hypertension with AS include common risk factors and the possibility that hypertension causes abnormally high tensile stress on the aortic leaflet. Nevertheless, age may act as the common factor, as both blood vessels stiffness and AS increase with age. Smoking is thought to be linked to dyslipidaemia and hypertension and as a promoter to atherosclerosis. Thus, the association that we found with the hazard of AS was expected. Although smoking at baseline was less common among those surviving longest, smoking associates with AS development in all the groups with follow-up ≥ 25 years.

Similarly, having atrial fibrillation during follow-up also associated with a later diagnosis of AS, which could be explained by shared common risk factors between AS and atrial fibrillation. The link between heredity for stroke in siblings and AS could be due to shared genetic and environmental factors leading to atherosclerosis and stroke in these families. Still, it could just as well be a chance finding. We also found an association between renal failure and the risk to develop AS, but only in the group followed 25–43 years. Renal failure seems to be a a risk factor for AS, as showed in the literature [34]. However, the wide confidence intervals in our study preclude firm conclusions as to the size of this effect.

## Strengths and limitations

Strengths of the present study include a large and relatively homogeneous study population, making it possible to eliminate some confounders. In addition, we had an extended follow-up (43 years), which is a strength in this rather slowly evolving disease. Yet, the study has some limitations. First, we used diagnostic codes instead of echocardiography and patients with potentially undiagnosed AS at baseline could have been included in the study. However, given that AS is unusual in men in their early 50 s, this is unlikely to have affected our results. The use of echocardiography in Gothenburg for the diagnosis of valve disease began in 1970, in addition to phonocardiography, pulse curves and heart catheterization used at this time, methods with low sensitivity that could have missed AS diagnosis. Even so, it is unlikely that surviving men with AS would not have been diagnosed at some point, given the characteristic heart murmur associated with AS. Second, because the information provided in a registry is limited, we could not describe the etiology or degree of severity of aortic valve morphology. We had no access to echocardiographic examinations and patient records could not be retrieved to a sufficient extent to validate diagnoses. Third, we could identify the outcome (i.e. AS) only in hospital patients because the NPR does not contain information about patients managed only in primary care. Fourth, we only studied middle-aged men and therefore our results cannot be generalized to women or older men.

## Conclusion

Our study showed that the cumulative incidence of AS was 3.2% in men followed from the age of 47–55 years and up to 43 years. Obesity, cholesterol level, hypertension, being a smoker or being diagnosed with atrial fibrillation during follow-up were associated with increased risk of developing AS. Considering that increasing longevity of the population leads to rising AS prevalence and that many of these factors are modifiable, a strategy delaying progression of aortic sclerosis and preventing significant AS might reduce the need for invasive aortic valvular intervention. Accordingly, multiple approaches targeting all known risk factors could reduce AS or delay its progression.

## Data Availability

Data are available from the sources stated in the paper on request to the data providers, fulfilling legal and regulatory requirements and with the permission from the Swedish Ethical Review Authority. Supporting data may be accessed if requested by contact with the authors.

## Abbreviations

AS: Aortic stenosis
BMI: Body mass index
BP: Blood pressure
CI: Confidence interval
DBP: Diastolic blood pressure
ICD: International Classification of Disease
IQR: Interquartile range
HR: Hazard ratio
NPR: Swedish National Patient Registry
PP: Pulse pressure
PPS: Primary Prevention Study
SBP: Systolic blood pressure
SEAS: Simvastatin Ezetimibe in AS
